# AI and Aphasia in the Digital Age: A Critical Review

**DOI:** 10.3390/brainsci14040383

**Published:** 2024-04-16

**Authors:** Adam John Privitera, Siew Hiang Sally Ng, Anthony Pak-Hin Kong, Brendan Stuart Weekes

**Affiliations:** 1 Centre for Research and Development in Learning, Nanyang Technological University, Singapore 637335, Singapore; sally.ngsh@ntu.edu.sg; 2 Institute for Pedagogical Innovation, Research, and Excellence, Nanyang Technological University, Singapore 637335, Singapore; 3 Academic Unit of Human Communication, Learning, and Development, The University of Hong Kong, Pokfulam, Hong Kong; akong@hku.hk; 4 Aphasia Research and Therapy (ART) Laboratory, The University of Hong Kong, Pokfulam, Hong Kong; 5 Faculty of Education, The University of Hong Kong, Pokfulam, Hong Kong; 6 Melbourne School of Psychological Sciences, University of Melbourne, Parkville 3010, Australia

**Keywords:** aphasia, artificial intelligence, deep learning, communication disorders, personalized therapy

## Abstract

Aphasiology has a long and rich tradition of contributing to understanding how culture, language, and social environment contribute to brain development and function. Recent breakthroughs in AI can transform the role of aphasiology in the digital age by leveraging speech data in all languages to model how damage to specific brain regions impacts linguistic universals such as grammar. These tools, including generative AI (ChatGPT) and natural language processing (NLP) models, could also inform practitioners working with clinical populations in the assessment and treatment of aphasia using AI-based interventions such as personalized therapy and adaptive platforms. Although these possibilities have generated enthusiasm in aphasiology, a rigorous interrogation of their limitations is necessary before AI is integrated into practice. We explain the history and first principles of reciprocity between AI and aphasiology, highlighting how lesioning neural networks opened the black box of cognitive neurolinguistic processing. We then argue that when more data from aphasia across languages become digitized and available online, deep learning will reveal hitherto unreported patterns of language processing of theoretical interest for aphasiologists. We also anticipate some problems using AI, including language biases, cultural, ethical, and scientific limitations, a misrepresentation of marginalized languages, and a lack of rigorous validation of tools. However, as these challenges are met with better governance, AI could have an equitable impact.

## 1. Introduction

The early 21st century has seen a revolution in healthcare. Clinicians and patients are now living in a digital age. Due to the sudden introduction of artificial intelligence (AI), including the now infamous ChatGPT, the diagnosis and treatment of aphasia have quickly adapted to the changes—but this has raised some concerns. In the public domain, we have witnessed a mix of hype and hysteria about the real power of AI. Headlines such as “Attack of the Psychochatbot”; “AI will replace us soon”; and “We don’t know what it means, but we’re scared” dominate the dinner table, care meetings, and tabloid newspapers. And with good reason. Several nations have banned ChatGPT and deep learning outright, including Russia, China (excluding Hong Kong), North Korea, Cuba, Iran, Italy (reversed), Syria, while others are considering doing so (Australia, Canada, and the USA). Accenture, Amazon, Apple, Samsung, Spotify, and several financial institutions in the European Union and the USA have also banned employees from using ChatGPT and other AI chatbot software systems. The government of the Philippines recently banned generative images, and others will soon follow. Identity theft, social engineering, and phishing attacks are cited as top concerns. AI image generators can also be misused to create false content that fuels the rapid spread of disinformation online. AI amplifies cultural biases in gender and race, leading to the reinforcement of stereotypes in media. Moving forward, it is vital for clinicians to understand these issues and to consider the impact of AI on their work.

The goal of this paper is to review the strengths and weaknesses of AI and deep learning and ask whether they offer solutions to aphasiologists. To do this, we take a critical position while acknowledging the real potential and resources that AI provides for understanding language, language learning, and language loss. We begin with a history of AI in aphasiology followed by an overview of issues and questions about AI in public discourse and in the scientific domain, and then focus on topics that are relevant to aphasiology. We begin with the premise that although AI is nascent, the roots of deep learning come from connectionism and so are not new in aphasia.

## 2. What Is Aphasia?

Aphasia is a language disorder caused by damage to specific brain areas responsible for language processing resulting in disrupted speaking, listening, reading, and writing. Aphasia does not affect intelligence, nor does it impact empathy, cultural awareness, introspection, learning, or the cognitive processes used to communicate non-verbally. Aphasia can be coincident with some cognitive impairments depending on pathology, residual fluency, and lesion location [1]. Stroke is the most common cause of aphasia, but head injuries, tumors, and neurological conditions such as dementia can also lead to aphasia. There are two major types of aphasia (i.e., fluent and non-fluent), of which each type includes different syndromes of aphasia. For example, Broca’s aphasia, also known as expressive aphasia, is a subtype of non-fluent aphasia characterized by difficulty producing speech. Patients with Broca’s aphasia often have trouble with syntax, finding the right words, and speaking in complete sentences. They may use short, simple sentences and struggle to form complex grammatical structures. Global aphasia is a severe form of non-fluent aphasia that affects all aspects of language processing, including speaking, understanding, reading, and writing. It is typically caused by extensive damage to the language centers in the brain, such as large lesions that affect multiple areas involved in language processing. Wernicke’s aphasia, also known as fluent or receptive aphasia, is characterized by difficulty understanding language. Patients with Wernicke’s aphasia often speak in long, grammatically correct sentences, but the content may be nonsensical or irrelevant. They may have difficulty recognizing words and understanding the meaning of sentences. Anomic aphasia is another fluent aphasia syndrome and is characterized by difficulty finding the right words. Patients with anomic aphasia may have trouble recalling the names of people or objects and often use filler words such as “thing” or “stuff” instead. They may also have difficulty with word retrieval and struggle to form coherent sentences. The condition of anomia is seen in virtually all people with aphasia (PWA), whereas Broca’s and Wernicke’s aphasia are typically associated with anterior and inferior lesions, respectively. It is important to note that these descriptions of different aphasia syndromes are not exhaustive and that the specific symptoms of aphasia vary depending on the type and severity of the lesion. However, understanding the specific manifestations of each type of aphasia can help healthcare professionals provide more targeted interventions and support for PWA.

Global prevalence rates of aphasia pose a significant public health challenge. While precise figures vary significantly across regions, it is estimated that tens of millions of individuals worldwide live with this condition [2]. Moreover, in light of ongoing demographic transitions characterized by increasing life expectancy and declining fertility rates, the global population is rapidly aging [3]. Given that age represents a significant risk factor for stroke and other cerebrovascular events, the number of PWA is projected to rise simultaneously with the aging demographic trend [4]. This demographic shift underscores an urgent need for comprehensive healthcare strategies to mitigate the growing burden of aphasia on individuals, families, and healthcare systems worldwide. The crisis has turned attention toward solutions from AI.

AI offers significant cost savings for healthcare, but these need to be offset by the societal costs. The financial and societal impact of aphasia worldwide is substantial. Changes in communication and daily functioning accompany aphasia, increasing healthcare utilization and costs, long-term care needs, and diminishing quality of life for PWA [5,6]. Significant indirect economic costs result via the loss of productivity and employment opportunities for PWA and their caregivers [7]. Societal repercussions also extend beyond PWA including reduced social participation, stigmatization, and impaired community integration [8]. These negative outcomes can, in part, be ameliorated via early detection and intervention [9]. Although findings related to the benefits of early intervention are mixed [10], there is no doubt that AI will reduce the costs of labor in the healthcare industry. Consequently, research to improve detection, categorization, and treatment in medicine using deep learning has accelerated. Although AI is a viable option for aphasia, there is virtually no research on the utility of deep learning in aphasiology. We invite scientist practitioners to consider AI for clinical work.

### 2.1. Aphasia Diagnosis and Treatment: Current Practices and Limits

Conventional methods for assessing and diagnosing aphasia typically involve a comprehensive evaluation by a speech–language pathologist (SLP) or a multidisciplinary team specializing in neurological disorders. Assessments include tests that are standardized for use in the native language, although translations of popular tests such as the Western Aphasia Battery WAB [11], the Boston Diagnostic Aphasia Examination BDAE [12], and the Comprehensive Aphasia Test CAT [13] are available. These tools are used to evaluate language abilities across modalities including comprehension, expression, repetition, naming, reading, and writing [14]. Simpler tests are used informally at the bedside and coincidentally are less sensitive to linguistic nuances, making them better suited to use with non-native speakers of a dominant language, e.g., a native Turkish speaker living in the UK [15,16]. Assessments such as the Token Test [17], the Copenhagen Cross-Linguistic Naming Test C-CLNT [15], and the Short Test for Aphasia [16] can be presented in a native language by an interpreter to assess communication skills without sociolinguistic biases. Neuroimaging techniques, such as magnetic resonance imaging (MRI) or computed tomography (CT) scans, are often utilized to identify the underlying brain damage or lesion associated with aphasia [18]. These methods play a role in accurately diagnosing aphasia, determining severity and characteristics, and guiding individualized treatment planning and intervention strategies as well as gaining an understanding of the neurobiological substrates of aphasia.

Although there is no cure for aphasia, a variety of different options that can significantly improve communication skills are presently available for PWA based on the severity and characteristics of their specific condition. Speech and language therapy, a cornerstone of aphasia treatment, focuses on improving language abilities through exercises targeting speaking, listening, reading, and writing skills [19]. Interventions may include repetition drills, communication strategies training, and computer-assisted therapy programs. Additionally, augmentative and alternative communication (AAC) devices such as communication boards or speech-generating devices can aid individuals with severe aphasia in expressing themselves [20]. Group therapy sessions can also provide opportunities for social interaction and support. Recent advancements in technology have also led to innovative treatments such as constraint-induced language therapy [21], showing promising results.

In addition to direct therapy, family and caregiver involvement is crucial for supporting PWA in their daily communication and rehabilitation efforts [22]. Education and training sessions for family members support them in understanding the challenges their loved ones face, and can aid in learning effective communication strategies to facilitate interaction [23]. Furthermore, interdisciplinary collaboration involving SLPs, neurologists, psychologists, and occupational therapists ensures comprehensive care addressing the multifaceted aspects of aphasia, including cognitive and emotional components [24]. The continuous monitoring and adjustment of treatment plans based on the individual’s progress and evolving needs contribute to optimizing outcomes and enhancing the quality of life for PWA [25,26].

### 2.2. Challenges in Understanding Aphasia across Cultures and Languages

While conventional methods for assessing and diagnosing aphasia provide valuable insights that can inform effective treatment, they also have notable limitations. Firstly, standardized language tests may not fully capture the diverse manifestations of aphasia, particularly in individuals with atypical or milder forms of the condition [27]. These standardized evaluations also often lack a detailed and objective assessment of post-sentence level performance such as spoken discourse [28,29]. Additionally, language assessments conducted in clinical settings may not always reflect the communication abilities of PWA in real-world contexts, where factors such as environmental demands, social interactions, and emotion play a significant role [30]. Cultural and linguistic diversity also present challenges in assessment, as standardized tests may not be culturally or linguistically appropriate for all PWA [15,16,31], which can negatively impact the effectiveness of treatment given the importance of identifying personalized care plans [32]. Further limits related to treatment exist where socioeconomic, geographic, and other barriers prevent PWA from accessing needed services that could positively impact their recovery and quality of life [33].

Addressing the long-term needs of PWA, including ongoing support for communication, social participation, and quality of life, poses a significant challenge that requires comprehensive and holistic approaches [34,35]. Therefore, while conventional methods have been essential in the diagnostic process for most of the 20th century, there is a need for a more comprehensive and individualized approach to assessment that considers a broader cultural context and the complexities of communication abilities in PWA within a social context. This issue was first investigated with scientific rigor by Bates and colleagues in the 1980s, including the birth of an Aphasic Language Data Exchange System ALDES [36,37]; Bates had the foresight to suggest that data sharing, neural networks, non-linear dynamics, and understanding the social factors that influence normal and impaired language are vital. Bates was also the first aphasiologist to suggest comparing PWA across languages with brain imaging.

Aphasiology researchers are indeed now much more interested in understanding the underlying linguistic mechanisms of aphasia at the neural level than during the time of Bates and colleagues, though see [38] for one earlier exception. Since brain damage varies in PWA, an idiosyncratic range of symptoms and presentations is expected within one syndrome and therefore at the brain neural level for each PWA [38]. PWA also present with considerable variability in recovery trajectories. This makes it difficult to develop theories of brain–behavior relationships in aphasia as well as generalizable treatment approaches [38,39]. Some PWA experience more severe impairments than others for a range of reasons, including pre-morbid individual differences, and recovery outcomes vary widely [38,39].

Researchers have found that level of education, literacy, lesion location, lesion, age, multilingualism, pre-existing cognitive abilities, and neuroplasticity explain some variability, guiding the selection of effective treatments for rehabilitation in PWA. Various approaches are available including pharmacotherapy and neuromodulation techniques like transcranial magnetic stimulation (TMS). However, their efficacy and optimal dosage are not yet known. Personalized treatment strategies tailored to individuals’ specific language impairments are needed. However, following PWA over extended periods requires resources and commitment from the client and therapist. AI offers an alternative by using big data to examine the co-variability of aphasia, outcomes, and therapy in PWA as a group. Similarly, differences in linguistic experience of PWA are critical for treatment planning but were not taken seriously in aphasiology until the beginning of this century. One reason is that established tests (in the hegemonic language of English) did not capture the nuance of communication difficulties in cross-linguistic aphasia. AI offers an alternative by using big data to translate languages instantly via speech-to-text, text-to-speech, and speech-to-speech AI. These developments require data sharing as well as a full representation of marginalized languages, and yet this is only now beginning to occur in aphasiology [40,41].

### 2.3. Aphasiology and Connectionism

Connectionism is a theoretical framework in cognitive science and AI that models mental or cognitive phenomena as interconnected networks of simple input–output computational units, often referred to as neurons or nodes. The history of connectionism can be found in several disciplines, including aphasiology, neuroscience, philosophy, psychology, and computer science. Ramón y Cajal’s research on the structure and function of the nervous system highlighted the role of neural connections, while Hebb’s theory of synaptic plasticity assumed that learning occurs through the strengthening or weakening of these connections. In early psychology, Thorndike proposed a theory of associationism to explain behavior and the mind. McCulloch and Pitts [42] first introduced the concept of an artificial neuron, a simplified mathematical model inspired by biological neurons. The McCulloch–Pitts model laid the foundation for computational models of neural networks. Rosenblatt defined the perceptron, a type of artificial neural network designed to perform binary classification tasks. A perceptron consists of a single layer of computational units (neurons) connected by weighted connections that learn to classify inputs into categories using a learning algorithm known as the perceptron learning rule. Connectionism then experienced a resurgence of interest in the 1980s, often called the connectionist or neural network revolution. Rumelhart, Hinton, and McClelland pioneered the development of parallel distributed processing (PDP) models [43], which are multilayer neural networks capable of learning complex patterns and representations, leading to the birth of connectionist neuropsychology, led by Plaut and Shallice, Coltheart and colleagues, Dell and colleagues, and Lambon Ralph, Patterson, Nickels, and Howard, who tested the outcome of “lesioning” a neural network by breaking connections at theoretically relevant locations to simulate a variety of aphasia syndromes; for reviews, see [39,44,45,46,47,48].

Rumelhart and colleagues [43] developed a PDP framework for modeling cognitive processes. PDP models use distributed representations and parallel processing to simulate various aspects of human cognition including perception, memory, and language processing. Researchers then developed learning algorithms for training neural networks, such as backpropagation, which enables multilayer networks to learn from labeled data and gradient descent optimization. Neural networks are computational models inspired by the structure and function of neurons. Examples include feedforward neural networks (FNNs), convolutional neural networks (CNNs), recurrent neural networks (RNNs), generative adversarial networks (GANs), autoencoders, recursive neural networks (RecNNs), attention mechanisms, and spiking neural networks (SNNs). Some features that are relevant to generative AI and NLP models are recurrent connections that allow models to maintain internal state or memory over time, pattern recognition to capture temporal relationships in sequential data, and the unsupervised learning of representations by reconstructing input data at the output layer. These models can be applied to a wide range of tasks in AI, including visual pattern recognition, speech recognition, NLP, and robotics. In cognitive science, models were adapted to simulate cognitive processes and inform theories of human cognition. Over the past decade, deep learning, a subfield of machine learning based on multilayer neural networks, has emerged as the dominant paradigm in AI research. Deep learning models (CNNs and RNNs) achieved success due to advances in computational resources, the availability of trillions of data, and algorithmic innovations; see reviews in [44,45,49]. Aphasiology played a reciprocal role in the development of connectionist models. Syndromes and even single cases were the motivation for developing models of speech, reading, and writing that were also assumed to explain unimpaired language processing and thus to have implications for competing accounts of cognitive processing including symbolic versus sub-symbolic accounts of language learning and language loss. This discipline lay dormant for years until the arrival of AI.

## 3. What Is AI?

AI refers to computer systems that perform tasks developed by human intelligence, e.g., learning, reasoning, problem solving, perception, speech recognition, and natural language understanding. AI developers aim to create software that emulates human cognitive functions and, in some cases, surpass human capabilities in domains such as art, education, health, medicine, robotics, security, and surveillance. Types of AI include machine learning (ML), which develops algorithms to learn from data in cyberspace. Unlike computer software that is programmed for a specific task, ML teaches itself. This is achieved by creating rules and configurations that emerge from patterns in big data collected over several decades and in a handful of dominant languages. For example, deep learning develops neural networks from image and speech data after learning the repetitive patterns harvested from code that is freely available, i.e., open-source AI algorithms designed to learn given one or two verbal prompts (words). NLP models allow deep learning to understand, interpret, and generate language output, and can be used in medical diagnosis, chatbots, language translation, and text analysis. NLP is the basis of ChatGPT. Computer vision enables machines to interpret and make decisions based on pictorial data without verbal input and can also be used for diagnoses as well as image or video recognition, object detection, face recognition, and vehicles. Robotics allows machines to perceive objects in the environment and to manipulate them, thus performing manual tasks needed in manufacturing, healthcare, and exploration. Expert systems are AI programs that mimic the decision-making abilities of a human expert in a specific domain by leveraging databases with “inference engines” to solve problems and provide advice, e.g., in virtual therapy wherein chatbots are tasked with providing counseling. Software adapted to healthcare and rehabilitation includes reinforcement learning using AI to make decisions in game playing, robotics, and speech therapy, all performed virtually; see review in [50].

AI has a rich and multilayered theoretical background. Information theory provides mathematical tools for quantifying information content, entropy, and uncertainty in AI. Graph theory is used to represent and analyze patterns, relationships, and structures in data. Algorithms from graph theory are employed in AI for tasks such as network analysis, social network modeling, recommendation systems, and NLP. Optimization theory uses linear programming and combinatorial optimization for ML, constraint satisfaction, planning, and game theory. Numerical methods can be used in optimization algorithms and simulation-based methods and for solving equation systems in computational intelligence and scientific computing. Control theory deals with the analysis and design of dynamic systems and feedback control mechanisms and is applied to AI for designing autonomous systems, robotics, adaptive control, and reinforcement learning. AI algorithms use mathematical principles including linear algebra, calculus, probability, and statistics. This is also a point where the reciprocal relationship between aphasiology and AI can be illustrated. Logistic regression is one example of a statistical method in AI and a simple form of a neural network. Coincidentally, logistic regression was first proposed to aphasiology researchers in London thirty years ago. Logistic regression uses a linear classification algorithm for binary classifications to model the relationship between independent variables and the performance of PWA using a logistic function called the sigmoid function that maps the input to a probability score between 0 and 1. Originally applied to the analysis of single case data using binary responses [39,51], then group data [52], logistic regression is now used to evaluate treatment efficacy, to identify risk factors, and recovery in aphasia and in other health fields. Although simple linear neural networks operate just like a logistic regression model, deep learning models capture complex non-linear relationships using big data and more sophisticated self-teaching algorithms.

### 3.1. Is AI Intelligent?

AI, deep learning, and ML are rapidly creating virtual experts in a variety of domains, impacting the lives of everyone worldwide. But what is the nature of their expertise? Are they intelligent? Psychologists divide AI into two categories: (1) narrow AI (weak AI) that is designed and trained for one task such as playing chess and voice and image recognition (including faces and speech), and (2) general AI (strong AI) to explain the ability to understand, learn, and apply knowledge across a wide range of tasks—similar to human intelligence. So far, only weak AI is functionally available.

Weak AI requires less intelligence than strong AI. For example, ML depends on classification of patterns in big data, reinforcement learning that connects these data to the output of the program, and training these associative connections to achieve a level of performance that is 100% correct. As with the learning of any skill, feedback and repetition are necessary. Human intelligence also requires these skills but supersedes the basic abilities of weak AI in several different ways. For example, humans can naturally empathize, imagine, intuit, reason, share subjective experience (called phenomenology), understand (comprehend and self-reflect), and have consciousness. Consciousness is a complex phenomenon that scientists have struggled to define and explain since the birth of humanity. Since we do not yet understand human consciousness, it cannot be classified as weak or strong. However, AI can be compared to many other cognitive abilities. Reasoning involves the manipulation of abstract symbols to solve a problem. The higher cognitive processes we need for communication, decision making, diagnosis, language, and speech therapy all require this skill and, critically, humans can generate verbal explanations of their own reasoning abilities. Some of the most popular AI-based applications can also do this, including ChatGPT, as it is simply a self-reporting question-answering algorithm. What it cannot do is “understand” the abstract symbols (words) it is using, and hence is not intelligent.

Some writers contend that AI does reflect on its own language and logic and is therefore capable of reasoning—at least at the verbal level [53]. Verbal refers to expression of concepts in speech, algebraic symbols, notations in music, song, and text. In non-verbal communication, abstract ideas are expressed without speech, signs, symbols, or text. However, the capability of AI has taught us that narrow intelligence is sufficient to generate verbal and non-verbal responses including images, sound, and text. Whether the responses can be considered intelligent is debated—but there is no doubt that even weak AI requires logic, mathematics, reasoning, and syntax. Note that, even if AI has weak intelligence, we can also measure AI against other human-specific criteria such as authenticity and truth.

An authentic and truthful agent aligns verbal output with core values and principles in different situations and requires self-awareness, i.e., understanding the values, beliefs, strengths, and weaknesses of the true self. Self-awareness can be measured culturally by acknowledging and respecting the cultural identity of the self in comparison with others. Authenticity is also associated with originality and the expression of a unique personal style. According to these criteria, generative AI might be considered authentic since it mimics sentient creatures. However, as authenticity depends on self-awareness, it is not at all authentic [54]. Generative AI contributes to the construction of personal and collective identity, e.g., virtual therapy, by producing output that reflects shared cultural values, traditions, and individual perspectives, and it evolves over time via deep learning. It can also be a source of inspiration, reflection, and exploration of human experience. However, it is not able to divorce fiction from truth, because it is not capable of authenticity or human judgment. This limit was clearly illustrated in the case of Google’s Gemini imagine generator, originally designed to increase diversity and reduce gender and racial biases in AI-generated images, which was withdrawn almost immediately after generating offensive and insensitive images of historic figures, including Nazi soldiers, as people of color [55].

### 3.2. Is AI Empathetic, Ethical, or Beneficent?

Empathy involves understanding and sharing the feelings, thoughts, and experiences of others. It is a complex emotional and cognitive ability that arises from our social and biological nature as humans. AI systems can be programmed to recognize and respond to human emotions through techniques such as sentiment analysis and affective computing, called “artificial empathy”. However, as they do not possess subjective experiences or emotions [56], they are not capable of empathy. AI algorithms can certainly analyze patterns in facial expressions, tone of voice, and text to infer emotions and tailor responses accordingly, but such responses are based on predetermined rules and statistical correlations and not subjective understanding or empathy. There have been efforts to develop AI systems to mimic empathy in interactions with users, such as therapy chatbots designed to provide emotional counseling or virtual assistants programmed to respond empathetically to users’ queries. However, these AI systems are limited as they lack authenticity and genuine emotional understanding. It is important to approach the development of AI with ethical, legal, and moral considerations in mind, so that systems are designed and deployed responsibly to respect human autonomy, emotions, and privacy [57].

AI systems often rely on large amounts of data to function effectively. However, the collection, storage, and analysis of personal data raises significant privacy concerns in healthcare. Protecting privacy rights and ensuring AI systems handle data responsibly are crucial ethical considerations. AI algorithms are highly complex and opaque, making it difficult to understand how they arrive at decisions. Ensuring transparency and holding developers and deployers accountable for their decisions and actions are essential for building trust and ensuring ethical behavior. However, as AI systems become more autonomous and capable of making decisions independently, questions will naturally arise about who is responsible for the outcomes. This requires better governance.

AI systems have the potential to cause harm if they malfunction, are hacked, or are used maliciously. AI is a tool, and like any tool, its ethical implications depend on how it is developed, deployed, and used. AI systems can inadvertently perpetuate and even exacerbate biases present in the data used to train them. For example, if historical data are used to train a hiring algorithm, but they also reflect biases against certain demographics, then the algorithm will perpetuate these biases by recommending candidates based on flawed criteria. Ensuring fairness and mitigating bias in AI is essential to promote equity and avoid discrimination. Maintaining human control over AI systems and ensuring they align with human values and goals are ethical considerations for aphasiology.

Beneficence is a concept in research ethics in which scientists consider participant’s welfare. Maleficence describes the practice that opposes the welfare of participants. AI has potential for maleficence, e.g., by disrupting industries, reshaping labor markets, and widening socioeconomic inequalities. To ensure the safety and security of AI, it is essential to prevent unintended consequences and malicious exploitation. Considerations for aphasiologists include ensuring that the benefits of AI are equitably distributed, addressing job displacement through retraining and social safety nets, and inclusive economic growth or equitable AI [58,59,60].

## 4. Collaboration with AI: Implications for Aphasia

The last decade has seen a rapid development in the methods used in contemporary AI. Originally focused on rules-based programming and statistical modeling, contemporary AI is increasingly based on complex neural networks and deep learning techniques as the availability of data grows [61]. This has enabled new generative capabilities with widespread applications including text and image generation using NLP [62,63]. As the capability of AI continues to expand, there is potential to address some pressing issues related to global health and wellbeing.

To date, a number of AI-based healthcare tools have been developed (reviewed in [50]), including analysis of large bodies of health-related data [64], informal diagnosis using patient-reported symptoms [65], physician diagnosis through image analysis [66,67], and assessment of treatment effects [68]. The validity of some of these tools in the domain of diagnosis is at least as accurate as medical experts [69]. AI is already proving to be beneficial.

Specific applications for AI in the diagnosis and treatment of aphasia have also been identified. In a recent scoping review, Azevedo and colleagues identified 28 published articles that investigated the use of deep learning in aphasia rehabilitation [70]. Of the identified studies, most focused on AI-based tools to diagnose or classify different syndromes of aphasia. The AI-guided classification of Broca’s, Wernicke’s, global, and anomic aphasia based on standardized language test performance (in English) was the most reported, e.g., [71], although studies using different categories of aphasia and different languages were also identified [72,73,74]. At present, only the hegemonic languages (e.g., English and Mandarin Chinese) offer enough data for AI harvesting from research studies. It is also notable that ever since the 1980s, there has been debate over the utility of syndrome-based group research studies. Bates and colleagues [36], who preferred group-based approaches, suggested that the distinction between fluent and non-fluent aphasia was likely to have more traction [1]. Others argue that single-case data are also useful in order to capture individual differences, e.g., premorbid linguistic background.

Most studies identified by Azevedo and colleagues [70] relied on the analysis of textual data transcribed after assessment by an SLP. However, two studies by Qin and colleagues utilized automatic speech recognition in their assessments to differentiate between aphasic and non-aphasic speech [72,73]. Of note is the observation that, with the exception of only two studies [75,76], models were trained using reported data taken from the Aachen Aphasia Test (AAT) dataset, and thus mostly in German [77,78], the English AphasiaBank [41], and the Cantonese AphasiaBank [40]. In our view, AI tools can have an impact on the inclusion of diagnoses across languages by reducing the resources associated with transcribing, translating, and coding patient speech samples in minority languages [79].

AI has also been applied to the analysis of imaging data collected from PWA. For example, Kristinsson et al. [80] used ML to predict aphasia severity and specific language measures based on a multimodal neuroimaging dataset. Neuroimaging data included task-based functional magnetic resonance imaging (fMRI), diffusion-based fractional anisotropy (FA) values, cerebral blood flow (CBF), and lesion load data. The WAB was used to measure aphasia severity and language functions. The results indicate that different neuroimaging modalities carry complementary information that can be integrated to more accurately depict how brain damage and the remaining functionality of intact brain tissue translate into language function in PWA, a finding achieved with the use of AI.

Fewer studies have explored the application of AI-based tools in the treatment of aphasia. Of these studies, most have focused on the use of automatic speech recognition (ASR) to augment or automate conventional forms of therapy, although the design of novel therapies has also been reported [81]. Tools using ASR systems have been developed to aid in the identification of aphasic speech during SLP-led progress monitoring [82], or independent participation in word-naming exercises [83]. The incorporation of ASR in AI-based tools provides significant advantages over previous efforts that necessitated the manual transcription of patient speech [84]. However, the status of the development of ASR varies considerably across languages, and problems with accuracy based on gender, racial background, and the use of regional or ethnic dialects have been reported [85,86]. In one case, an investigation of the Microsoft Speech Services ASR identified systematically higher error rates for African American, Chicanx, and Native American English speakers relative to Caucasian English speakers sampled from the same geographic region [86]. Furthermore, despite promising results, a significant limit associated with ASR systems is that patients experience issues with usability due to reliance on speech. This limitation is likely to be acutely experienced by patients who have progressive forms of aphasia where symptom severity increases over time [87], or those in the early (recovery) stage of aphasia when PWA produce a very limited amount of spontaneous speech output.

As the capabilities of AI continue to expand, its potential to support the diagnosis and treatment of aphasia looks promising. However, as with any technological development, advances in utility will not be equally shared by all groups. Considering that previous studies have identified significant barriers to the adoption of AI-based tools within specific linguistic populations, e.g., [85,86], there is an ethical imperative to proactively identify an equitable path forward. This is especially critical given the positive impact these tools could have on PWA who currently lack access to aphasiology services due to linguistic barriers and poverty. Furthermore, because the introduction of AI-based tools into professional practice has significant implications for how current and future SLPs are trained and upskilled, there is a need to consider how these tools should be integrated into training programs. Finally, although research findings have been summarized in previous reviews, there have been very few open debates or discussions in aphasiology as to the ethical considerations inherent in use of AI tools.

## 5. Practical Considerations in the Equitable Rollout of AI

The integration of AI in the assessment and treatment of aphasia presents a promising frontier with potential to revolutionize therapeutic outcomes. While AI-driven applications can offer personalized therapy and the monitoring of progress for PWA, several technical limits and ethical issues may hinder the scaling and equitable implementation of these solutions. First, extensive data requirements for training AI models generally require large volumes of high-quality, annotated linguistic data to develop algorithms capable of understanding and generating human language [88]. This is a challenging and resource-intensive task to undertake. Major considerations regarding data requirements for training an AI model for aphasia diagnosis include (1) data quantity and diversity, (2) data quality and annotation, and (3) data bias and ethics.

The production of an AI model to handle complex tasks like aphasia diagnosis and therapy requires tens to hundreds of thousands of labeled speech samples for training [89]. The diversity of aphasia symptoms and the individual variability among patients necessitate a dataset that is vast. Additionally, training an accurate and reliable model requires that speech samples are clear and of high quality. The preprocessing of data to remove noise and segment them for feature extraction is necessary [90]. To accurately transcribe and label speech samples for aphasia diagnosis is a complex and time-consuming process requiring expertise from SLPs, neurologists, and other professionals. AI systems often struggle to generalize beyond the specific tasks and datasets they were trained on, limiting their ability to adapt to new environments or tasks. Achieving robust generalization and transfer of learning capabilities across diverse domains and datasets is a key scientific challenge in AI research.

Concerns around data bias and ethics are less straightforward than those related to data quantity and quality. To prevent inherent latent biases and discrimination of the AI model developed, deliberate care must be taken to ensure the inclusion of speech samples from a diverse profile of patients and representative of the spectrum of language impairments [91]. This would include, at a minimum, a diverse range of samples from patients with different types of aphasia and varying degrees of severity. Additionally, samples should be provided across a wide range of ethnic and linguistic backgrounds to proactively address issues identified in the development of related ASR systems [85]. Further complicating data requirements is the need for patient data privacy and security as linguistic data used in healthcare applications are sensitive and subject to strict regulations like the General Data Protection Regulation (GDPR) in the European Union [92]. Compliance with these regulations can limit the availability of data for AI training, potentially hindering the development of robust, effective, and equitable models for aphasia diagnosis and treatment.

Additional hurdles to the equitable rollout of AI in the assessment and treatment of aphasia relate to technological costs and availability. To fuel the training and implementation of such a complex AI-driven healthcare application, a substantial expense budget must be available to grow the computational power of hardware, particularly to increase the number of graphics processing units (GPUs) to handle the complexity of these AI models and to handle the real-time processing of large amounts of data [93]. Supporting remote diagnosis necessitates the availability of a high-speed network communication infrastructure for real-time data transmission between devices and servers. The current network infrastructures in most healthcare facilities are not designed to handle the massive amount of synchronous data transfer that deep learning requires [94], which may result in issues such as excessive latency.

Finally, personalized diagnosis and monitoring requires investment in wearable healthcare technologies to collect and send real-time source data to centralized processing servers [95]. Frequent communication to acquire data would inadvertently increase the burden on the network bandwidth and put pressure on demands to improve the existing storage infrastructure. In addition, it may also pose potential security risks. Measures to store and transmit data securely can further drive up the cost of adopting such AI-driven solutions. While healthcare institutions in developed countries with high-speed internet ecosystems may overcome this increased hardware, data storage, and internet network demand if they have the financial means, healthcare institutions in developing countries with low internet penetration rates in low-resource settings where the technological literacy level of users is low [96] may find such AI-driven solutions to be infeasible.

## 6. Ethical Considerations

### 6.1. Establishment of Ethical Guidelines

Researchers are exploring ethical frameworks and guidelines for AI that promote cultural diversity, inclusion, and equity. A code of ethics for responsible conduct in healthcare is desirable. Codes of ethics, like the one provided by the National Health Service (NHS) UK, play a role in promoting responsible conduct within the healthcare industry. The code establishes a clear set of principles and guidelines for ethical behavior. This provides a benchmark for all professionals to navigate complex situations and make responsible decisions. By outlining core values like honesty, trustworthiness, and respect for privacy, the code sets expectations for how allied health professionals should interact with clients, colleagues, and the public. A code of ethics fosters public trust in a profession. When professionals adhere to ethical principles, the public is more likely to feel confident sharing their data and engaging with technology. This public trust is essential for the continued growth and adoption of Information and Communications Technology (ICT) solutions in various sectors like healthcare, finance, and government services. The healthcare industry faces unique ethical challenges, such as data privacy concerns, algorithmic bias, and cybersecurity threats. A code provides a framework for considering these issues and making ethically sound choices. By offering guidance on handling data responsibly, mitigating security risks, and avoiding conflicts of interest, the code helps allied health professionals navigate complex situations. A code establishes a mechanism for holding professionals accountable for their actions. If a professional breaches the code, then they might face disciplinary action from the NHS or their employer. This accountability system discourages unethical behavior and encourages professionals to uphold the highest ethical standards. Codes of ethics are living documents that evolve alongside the ever-changing technological landscape. The NHS Code of Ethics is reviewed and updated periodically to reflect new challenges and emerging ethical considerations within the ICT industry. This ongoing process ensures the code remains relevant and continues to guide responsible conduct in a dynamic field.

### 6.2. Who Benefits from AI?

There are ethical concerns regarding the lack of compensation and benefit sharing for those who contribute their data or labor to train AI models. Whittaker et al. [97] argued that the current AI ecosystem encourages an “extraction model” where AI companies profit from the contribution without paying due remuneration and acknowledgement to individuals for their “data labor”. This raises questions about the power imbalance between AI companies, data contributors, moderators, and annotators [98]. Furthermore, the eventual AI systems developed may also not be accessible or benefit these individuals who have contributed their personal data to train the model. For example, the AI system developed by Google Health for detecting breast cancer was trained using a dataset containing 90,000 mammogram images and associated data from a diverse pool of patients including those from underserved communities across the UK and USA. However, the deployment of this system is concentrated in well-funded hospitals and medical centers that can afford to implement such advanced technologies [99]. There are other similar examples where the resulting AI healthcare systems become unaffordable and inaccessible to the patient population whose data are used to train the system [100,101]. These examples highlight ethical concerns regarding equitable benefit sharing from the development of AI healthcare systems, despite relying on data contribution from diverse patient populations. It is important to address these ethical issues of fair compensation and benefit sharing to ensure the responsible and equitable development of AI healthcare systems.

### 6.3. Environmental Impact

Discussions of the promises that AI brings to humankind often overshadow the potential negative impact it has on our environment. AI systems, especially ML and deep learning systems, require immense computational power. Data centers required to house AI infrastructure are already consuming around 1% of global electricity [102]. As AI models become increasingly larger and complex, energy consumption is expected to increase exponentially. According to a study by Strubell et al. [103], training a single AI model can emit as much carbon dioxide as the lifetime emissions of five cars. The energy-intensive nature of developing and sustaining these AI systems contributes significantly to greenhouse gas emissions and environmental degradation. Furthermore, the rapid development cycles and constant hardware upgrades lead to increased electronic waste—one of the fastest growing waste streams in the world. By 2030, it is estimated that the annual e-waste production will reach a staggering 75 million metric tons [104]. The increased demand and extraction of rare earth minerals for AI hardware manufacturing is also expected to cause much environmental damage [105]. Addressing these environmental concerns will require concerted efforts from researchers, developers, and policy makers to prioritize energy efficiency and sustainable practices in the development and deployment of AI systems [106].

## 7. Clinical Considerations

The use of AI tools in the assessment and treatment of aphasia has the potential to revolutionize the field of speech–language pathology. Using generative AI tools has the potential to provide unique opportunities for clinicians to both assess and treat PWA. For example, tools such as NLP algorithms can help clinicians to better understand the specific language deficits that PWA may be experiencing. These tools can analyze large amounts of data to identify patterns in language use and processing, which may offer complementary information for clinicians to develop targeted treatment plans for individual patients. One way that clinical data can be used to improve the quality of generative AI tools is through the process of ML, which typically involves the use of algorithms to analyze large amounts of data and identify patterns or trends. In the case of generative AI tools for aphasia, ML can be used to analyze clinical data (such as language samples, linguistic and cognitive assessment results, neuroimaging data, and treatment progress data) collected from PWA. These data can be used to train algorithms to recognize specific language deficits and patterns of language use associated with different degrees and/or types of aphasia. In theory, as more clinical data are collected and analyzed, these algorithms can become more accurate and effective in identifying language deficits and developing personalized treatment plans for individual patients, potentially leading to improved clinical outcomes and a more efficient use of resources in the management of aphasia. 

However, there are also several challenges to consider when using AI tools in a clinical setting. One such challenge is the need to ensure that AI algorithms are developed and implemented ethically and without bias. To effectively use generative AI tools in the assessment and treatment of aphasia, SLPs should have a thorough understanding of both the technology and the unique needs of their PWA clients. A major challenge in AI is making complex models understandable and interpretable. Deep learning models, in particular, are often seen as “black boxes” because they lack transparency in how they arrive at their decisions. Developing techniques to explain AI models’ reasoning and decision-making processes is crucial for meeting professional values such as trust, accountability, and acceptance for critical applications in healthcare practice and research. In addition to technical guidance, SLPs should also receive training on how to effectively integrate AI tools into their clinical practice. This includes understanding how to collect and analyze clinically meaningful data using AI tools, and how to use these data to develop more personalized and effective treatment plans for PWAs. Similar to the conventional, traditional approach of aphasia management, it is important for SLPs to maintain a patient-centered approach when working with generative AI tools. It is crucial that SLPs effectively communicate with PWA and their families about the use of AI tools in their assessment and treatment and ensure that these stakeholders are comfortable with the use of this technology. As such, SLPs can balance the use of AI and human-based intervention with the importance of maintaining a strong clinician–patient relationship. Finally, privacy and data security must be carefully managed to ensure that PWA data are protected and used only for appropriate purposes. Clinicians must ensure that the development and use of AI tools is transparent and accountable, and that PWA understand how their data are being used.

### AI as a Virtual Therapist

The use of AI as a “virtual therapist” raises significant concerns. PWA suffer not only from the characteristic language deficits, but also often experience social isolation and emotional distress [107]. While AI-powered virtual therapists could potentially provide accessible and convenient support, there are valid concerns about their ability to truly understand and respond to the complex emotional needs of these individuals. One major consideration is the potential for AI therapists to lack the empathy and nuanced understanding that human therapists possess. While AI systems can be programmed to recognize and respond to human emotions through techniques such as sentiment analysis and affective computing, they do not possess subjective experiences or emotions themselves. AI algorithms can analyze patterns in facial expressions, tone of voice, and text to infer emotions and tailor responses accordingly, but responses are based on predetermined rules and statistical correlations rather than genuine understanding or empathy.

Aphasia is a highly individualized condition, and each person’s experience with it is unique [8]. An AI system, no matter how advanced, may struggle to comprehend the intricate emotional and psychological aspects of this disorder. Previously developed empathetic AI systems are limited in their ability to truly comprehend and empathize with human emotions, as they lack consciousness, subjective experiences, and genuine emotional understanding. While AI technology continues to advance rapidly, achieving empathy in machines remains a distant goal. It is important to approach AI development with these considerations in mind, ensuring that AI systems are designed and deployed responsibly to respect human emotions, privacy, and autonomy. The risk of providing impersonal or insensitive responses could further exacerbate feelings of isolation and distress for those seeking support, as highlighted by research on the importance of empathy in therapy [108].

Furthermore, issues of privacy and data security arise when discussing sensitive personal information with an AI system. The very nature of sharing intimate details with an AI system brings to the forefront the apprehension surrounding potential misuse or unauthorized access to such data. It underscores the need for implementing robust data protection measures and fostering transparent policies. Ethical guidelines for AI in healthcare, as outlined by Char et al. [109], emphasize the critical importance of addressing these concerns. One of the primary objectives is to establish a framework that not only safeguards individuals’ privacy but also ensures the integrity and confidentiality of their personal data. This entails deploying state-of-the-art encryption protocols, access controls, and other technical safeguards to mitigate the risk of data breaches or unauthorized access. Moreover, transparency is paramount in building trust between users and virtual therapists. Users must be informed about how their data are collected, stored, and utilized, as well as the purposes for which it is being used. This transparency empowers individuals to make informed decisions about their engagement with AI therapists and enables them to exercise greater control over their personal information. Finally, ongoing monitoring and auditing mechanisms must be put in place to ensure compliance with data protection regulations and ethical standards. Regular assessments of data handling practices can help identify potential vulnerabilities and areas for improvement, thereby bolstering the overall security posture of AI-driven therapeutic platforms.

While AI-powered virtual therapists could potentially improve access to supportive services for PWA, it is crucial to carefully weigh the ethical implications and prioritize the well-being and dignity of these individuals. Ongoing research, ethical guidelines, and collaboration with healthcare professionals and PWA are necessary to ensure that any implementation of AI in this context is done responsibly and with the utmost consideration for the unique needs of this population [110].

## 8. Concluding Remarks

Chomsky argues that generative AI is of no interest to understanding the human mind or brain [111]. His most biting criticism is that AI cannot distinguish facts that exist in the real world (truth) from facts that are generated about a fictional world (lies) based on data available in cyberspace [112]. To summarize, because AI models lack self-awareness, cannot recognize authenticity, and cannot discriminate between fact and fiction subjectively, they lack utility beyond what human cognition allows. Such abilities require a theory of mind, mental models of how we predict our immediate future, and reasoning processes that can derive testable predictions about the environment we enter every time we step into the world. As to the future, enthusiasts of AI express optimism arguing that present subjective diagnostic methods are imperfect. Expertise, knowledge, and wisdom will be usurped by AI via permanent, accessible, and renewing sources of facts that require no replication or validation, as is the hallmark of scientific methods. AI skeptics on the other hand express pessimism. At present, accountability in data sharing is absent and often occurs without the consent of PWA, the clinician, or even the institution; for an early warning of this, see [36]. Training data are biased, perhaps more so than human clinicians who have years of experience. It is also likely that cost-effective, expeditious AI will lead to the deskilling of professionals and “privileging” of those who can afford technology. Ethical questions can only be addressed with governance allowing malfeasance, e.g., monetizing data for corporate interests, to be interrogated. The specificity and clinical validity of AI diagnosis and treatment in aphasiology is thus still open to question. However, translational gaps between AI output and clinical efficacy are now emerging and require rigorous new scientific research; see review in [113]. We contend that AI is irresistible and unstoppable. However, the voices of aphasiologists are unheard at best and ignored at worst. Given that the science of aphasiology has had a pivotal role in the development of AI over the past 50 years and has a critical stake in the equitable and ethical use of AI, we need a platform to express our views and to monitor developments at a policy level. We therefore recommend that lay, professional, and scientific bodies respond urgently.
